# Integrated bioinformatics analysis to develop diagnostic models for malignant transformation of chronic proliferative diseases

**DOI:** 10.1097/BS9.0000000000000226

**Published:** 2025-04-07

**Authors:** Hua Liu, Sheng Lin, Pei-Xuan Chen, Juan Min, Xia-Yang Liu, Ting Guan, Chao-Ying Yang, Xiao-Juan Xiao, De-Hui Xiong, Sheng-Jie Sun, Ling Nie, Han Gong, Xu-Sheng Wu, Xiao-Feng He, Jing Liu

**Affiliations:** a Shenzhen Health Development Research and Data Management Center, Shenzhen 518028, China; b Molecular Biology Research Center, School of Life Sciences, Hunan Province Key Laboratory of Basic and Applied Hematology, Central South University, Changsha 410013, China; c Department of Hematology, Xiangya Hospital, Central South University, Changsha 410078, China

**Keywords:** Acute myeloid leukemia, Bioinformatics analysis, Biomarker, Hub genes, Machine learning, Polycythemia vera

## Abstract

The combined analysis of dual diseases can provide new insights into pathogenic mechanisms, identify novel biomarkers, and develop targeted therapeutic strategies. Polycythemia vera (PV) is a chronic myeloproliferative neoplasm associated with a risk of acute myeloid leukemia (AML) transformation. However, the chronic nature of disease transformation complicates longitudinal high-throughput sequencing studies of patients with PV before and after AML transformation. This study aimed to develop a diagnostic model for malignant transformation of chronic proliferative diseases, addressing the challenges of early detection and intervention. Integrated public datasets of PV and AML were analyzed to identify differentially expressed genes (DEGs) and construct a weighted correlation network. Machine-learning algorithms screen genes for potential biomarkers, leading to the development of diagnostic models. Clinical specimens were collected to validate gene expression. cMAP and molecular docking predicted potential drugs. In vitro experiments were performed to assess drug efficacy in PV and AML cells. CIBERSORT and single-cell RNA-sequencing (scRNA-seq) analyses were used to explore the impact of hub genes on the tumor microenvironment. We identified 24 genes shared between PV and AML, which were enriched in immune-related pathways. Lactoferrin (LTF) and G protein-coupled receptor 65 (GPR65) were integrated into a nomogram with a robust predictive power. The predicted drug vemurafenib inhibited proliferation and increased apoptosis in PV and AML cells. TME analysis has linked these biomarkers to macrophages. Clinical samples were used to confirm LTF and GPR65 expression levels. We identified shared genes between PV and AML and developed a diagnostic nomogram that offers a novel avenue for the diagnosis and clinical management of AML-related PV.

## 1. INTRODUCTION

Recent advancements in sequencing technology and the application of multi-omics have positioned bioinformatic analysis as a crucial component of biomedical research. The emerging trend in multi-disease analysis offers novel insights into the pathogenic mechanisms shared across different disorders.^1–5^ Zhu et al^2^ used weighted gene co-expression network analysis (WGCNA) and other methodologies to identify 4 hub genes that play pivotal roles in autism spectrum disorder and inflammatory bowel disease. Similarly, Dai et al^6^ demonstrated the significance of mitochondrial dysfunction in severe acute respiratory syndrome coronavirus 2 (SARS-CoV-2) infection and cardiovascular disease through protein–protein interaction (PPI) and immune microenvironment analyses. These studies underscore the value of integrating multiple bioinformatic approaches for comprehensive analysis, revealing cross-disease molecular interactions, identifying shared biomarkers, and elucidating intrinsic biological connections. These integrated approaches are promising for enhancing disease diagnosis and for developing targeted therapeutic strategies.

Polycythemia vera (PV) is a chronic myeloproliferative neoplasm (MPN) characterized by the excessive production of red blood cells, white blood cells, and platelets in the bone marrow. The JAK2 V617F mutation is prevalent in almost all patients with PV and plays a pivotal role in hematopoietic disorders.^7^ Acute myeloid leukemia (AML) is the predominant form of acute leukemia in adults and is characterized by a decline in healthy hematopoietic cells and extensive proliferation of blast cells.^8^ AML is a type of cancer characterized by significant genetic diversity, high mortality, and a 5-year survival rate below the 50% threshold.^9^ Although the median survival time of patients with PV infection exceeds 10 years, the natural course of the disease may be disrupted by leukemic events. Current treatment approaches targeting PV have yet to demonstrate efficacy in prolonging survival or mitigating the risk of leukemia progression.^10^ A comprehensive study of Swedish patients with MPNs revealed those with AML transformation exhibited a median survival of only 3 months from the date of AML diagnosis, with the majority having a prior PV diagnosis.^11^ Transformation of PV into AML poses a significant clinical challenge; however, the molecular mechanisms underlying this evolution remain unclear. The protracted nature of disease transformation complicates longitudinal high-throughput sequencing studies of patients with PV before and after AML transformation.

This study aimed to delve deeper into the molecular profiles of patients with PV and AML using bioinformatic methods to uncover shared molecular signaling pathways and diagnostic biomarkers between PV and AML. By integrating these data, we aimed to identify novel diagnostic and therapeutic strategies specifically tailored to patients with PV progressing to AML. Our comprehensive analysis developed a diagnostic model applicable to both disorders, addressing the critical need for improved disease management.

## 2. MATERIALS AND METHODS

### 2.1. Data collection

Two PV datasets (GSE54644 and GSE103237) were obtained from the GEO database (https://www.ncbi.nlm.nih.gov/geo/). The SVA package^12^ was used to debatch and integrate the 2 PV datasets. The TCGA-AML cohort and normal control data were retrieved from the UCSC Xena database (https://xena.ucsc.edu/). Two supplementary AML datasets (GSE30029 and GSE37307) were downloaded from the GEO database. An external independent PV cohort (GSE47018) was obtained from the GEO database. Table S1, http://links.lww.com/BS/A110, explains the datasets used in this study.

### 2.2. Identification of key genes in PV and AML

The limma package^13^ was used to identify DEGs in PV or AML, using thresholds of *P* value <.05 and |log2(fold change)| > 0.5. The WGCNA algorithm^14^ was used to identify key gene modules associated with PV or AML. Specifically, the top 5000 genes determined by the largest median absolute deviation were filtered. The optimal “soft” threshold power (*β*) for constructing scale-free networks was set to 8, with other settings maintained at default values. The top 3 co-expressed modules were selected as key modules for PV or AML. Subsequently, we conducted an intersection analysis between the DEGs identified by limma and the key gene modules identified by WGCNA to identify key shared genes in PV and AML.

### 2.3. Comprehensive analysis of shared genes in PV and AML

To elucidate the interactions between shared genes in PV and AML, we constructed and visualized a PPI network using the GeneMANIA database.^15^ Gene Ontology (GO)^16^ and Kyoto Encyclopedia of Genes and Genomes (KEGG)^17^ enrichment analyses were performed to explore the biological processes (BPs) associated with these shared genes. In addition, a connectivity map (cMAP) analysis was conducted using the Broad Institute’s L1000 database^18^ to investigate potential drug compounds for AML-associated PV and to identify the top 10 small-molecule connectivity scores.

### 2.4. Construction evaluation of a nomogram model

To identify biomarkers in PV and AML, we used 4 machine-learning methods (LASSO, random forest [RF], SVM-RFE, and XGBoost) to identify robust candidates. The key parameter settings for the machine-learning algorithms used in this study are as follows. For the LASSO algorithm, the parameters include α = 1 (indicating regularization strength) and family = “binomial” (specifying the response variable type as binomial), and xvar = “lambda” defines the cross-validation parameters. In the RF algorithm, max_features = “auto” determines the number of features considered for splitting at each node, whereas nfold = 5 specifies the number of cross-validation folds. SVM-RFE uses *k* = 10 (indicating the number of features selected at each iteration), halve above = 100 to control the halving threshold, and nfold = 5 for cross-validation. Finally, the XGBoost algorithm uses eval_metric = “logloss” to evaluate the performance, η = 0.1, to set the learning rate, and max_depth = 3 to limit the depth of the tree to control the model complexity. Subsequently, the regplot package was used to construct a diagnostic nomogram model to predict AML-associated PV. Nomogram performance was assessed by plotting the area under the receiver operating characteristic (ROC) curve. Calibration and decision curve analyses (DCA) were performed to evaluate the predictive accuracy of the nomogram. The diagnostic model was validated using the external dataset, GSE47018.

### 2.5. Immune infiltration analysis

The CIBERSORT algorithm^19^ was used to evaluate the proportions of 22 immune cells in the PV based on gene expression profiles, the CIBERSORT algorithm^19^ was used. Spearman analysis was conducted to estimate the correlation between the hub genes and lymphocytes.

### 2.6. Reverse transcription-quantitative polymerase chain reaction (RT-qPCR)

RT-qPCR was performed as previously described.^20,21^ Primers specific to the 2-gene panel of lactoferrin (LTF) and G protein-coupled receptor 65 (GPR65) were designed as follows:

LTF:

Forward primer: AGAGCCTTCGTTTGCCAAGT

Reverse primer: ACAGGTCGCAGTTTGTAGGG

GPR65:

Forward primer: GGTGAGGGGGAAGTGATGTG

Reverse primer: GAGGTCCCGTACAGAACACG

### 2.7. Proliferation assay

For the cell proliferation assay, 20 μL of methyl thiazolyl tetrazolium (MTT) (5 g/L, Sigma, Beijing, China) was added to each well and incubated at 37°C for 4 hours. Subsequently, 150 μL of dimethyl sulfoxide (DMSO; Sigma) was added to dissolve the formazan crystals. The plate was then covered with aluminum foil and shaken for 15 minutes. Cell proliferation was quantified by measuring absorbance at 450 nm using a spectrophotometric microplate reader.

### 2.8. Flow cytometry

For apoptosis analysis, the cells were collected, washed with phosphate buffer saline (PBS), and stained with propidium iodide (PI)/fluorescein isothiocyanate (FITC). The stained cells were incubated at 37°C in the dark for 30 minutes. Apoptosis was detected using flow cytometry.

### 2.9. Statistical analysis

All statistical analyses and visualizations were performed using the R software (version 4.2.1) and GraphPad Prism software (version 8.0.1). The Mann–Whitney *U* test was used to compare differences between the different subgroups. All *P* values were 2-sided, and statistical significance was defined as *P <* .05, unless otherwise specified.

## 3. RESULTS

### 3.1. Integration of PV cohorts and identification of key genes

**Figure 1.**
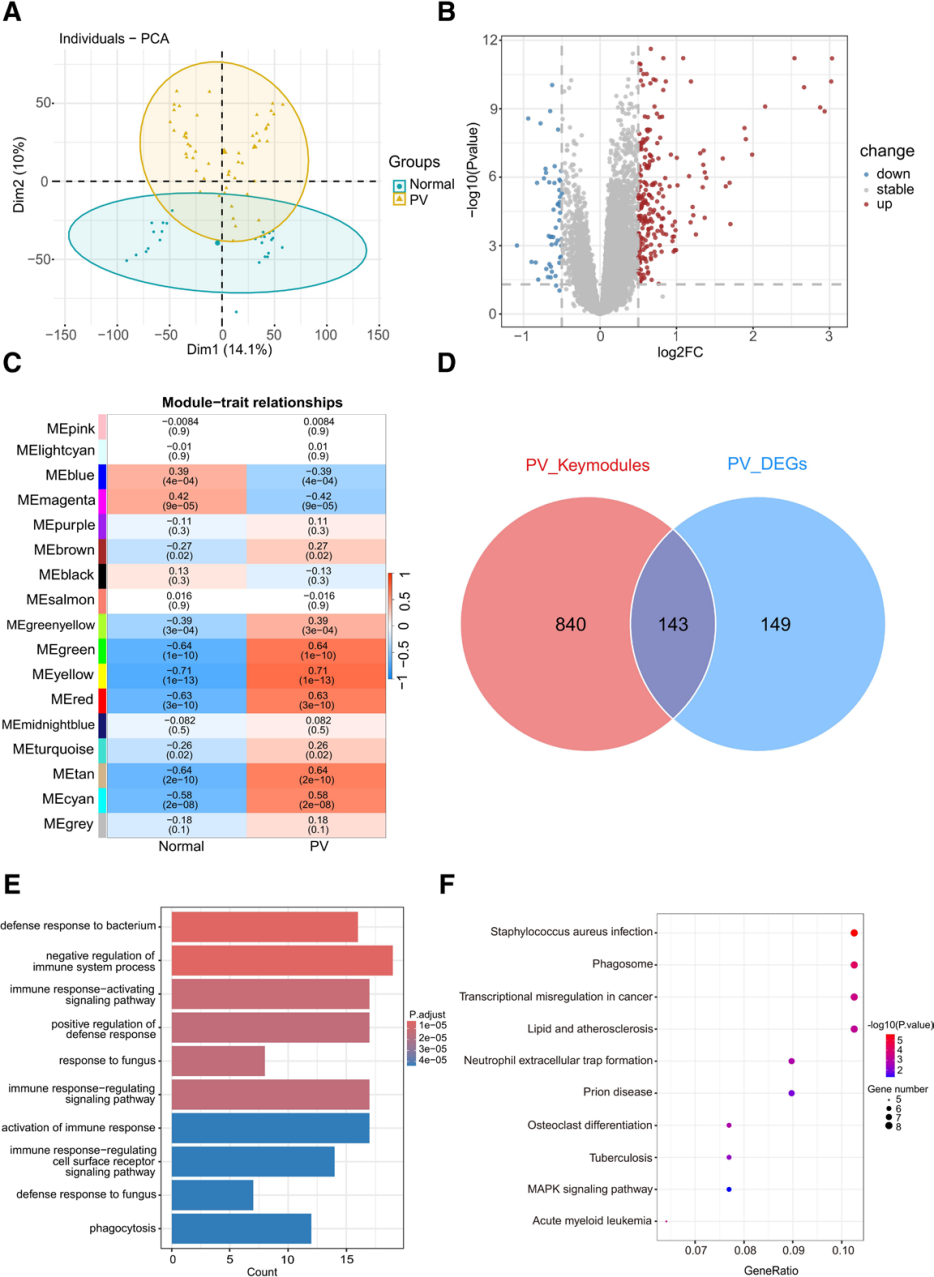
Integration of PV datasets and differential expression analysis. (A) PCA plot displaying the distribution of PV patients and normal samples after the removal of batch effects. (B) Volcano plot presenting the differential expression analysis results, showcasing the DEGs between PV patients and normal samples. Upregulated genes are represented by red dots, while downregulated genes are represented by blue dots. (C) Heatmap illustrating the relationship between co-expression modules and disease status. The correlation between model Eigen genes and PV traits (top) is depicted, along with corresponding *P* values (bottom). (D) Venn diagram depicting the intersection of key modules and DEGs. A total of 143 key genes in PV were identified. (E, F) The GO (E) and KEGG (F) results for key genes of PV. DEG = differentially expressed gene, GO = gene ontology, KEGG = Kyoto Encyclopedia of Genes and Genomes, PCA = principal component analysis, PV = polycythemia vera.

First, we integrated 2 PV datasets. The PCA plot highlighted significantly distinct distributions between patients with PV and healthy controls after batch-effect removal (**Fig. 1A**). Differential analysis revealed 241 upregulated and 49 downregulated DEGs in the PV group (**Fig. 1B**). To further explore the co-expressed modules that were highly correlated with PV, the WGCNA algorithm was used. Based on the scale-free topology criterion, the automatic network construction algorithm determines a soft-thresholding power of *β* = 8 as the optimal power for network construction. The heatmap depicts the correlation coefficient and *P* value of each module co-expressed with the PV (**Fig. 1C**). The top 3 modules exhibiting the highest correlation were the yellow module (548 genes, *r* = 0.71, *P* = 1e-13), green module (363 genes, *r* = 0.64, *P* = 1e-10), and tan module (72 genes, *r* = 0.64, *P* = 2e-10). Intersection analysis between the DEGs and genes identified by WGCNA yielded 143 key PV genes for further analysis (**Fig. 1D**; Table S1, http://links.lww.com/BS/A110). GO and KEGG analyses were conducted to explore the biological functions of these key genes. In the GO analysis, the top 3 terms were defense response to bacteria, negative regulation of the immune system process, and immune response-activating signaling pathway (**Fig. 1E**, Table S3, http://links.lww.com/BS/A112). KEGG analysis of key PV genes indicated associations with *Staphylococcus aureus* infection, phagosomes, and transcriptional dysregulation in cancer (**Fig. 1F**, Table S4, http://links.lww.com/BS/A113). The KEGG results included the term “Acute Myeloid Leukemia,” emphasizing the potential functional integration of the 2 diseases.

### 3.2. Screening of key genes related to AML

**Figure 2.**
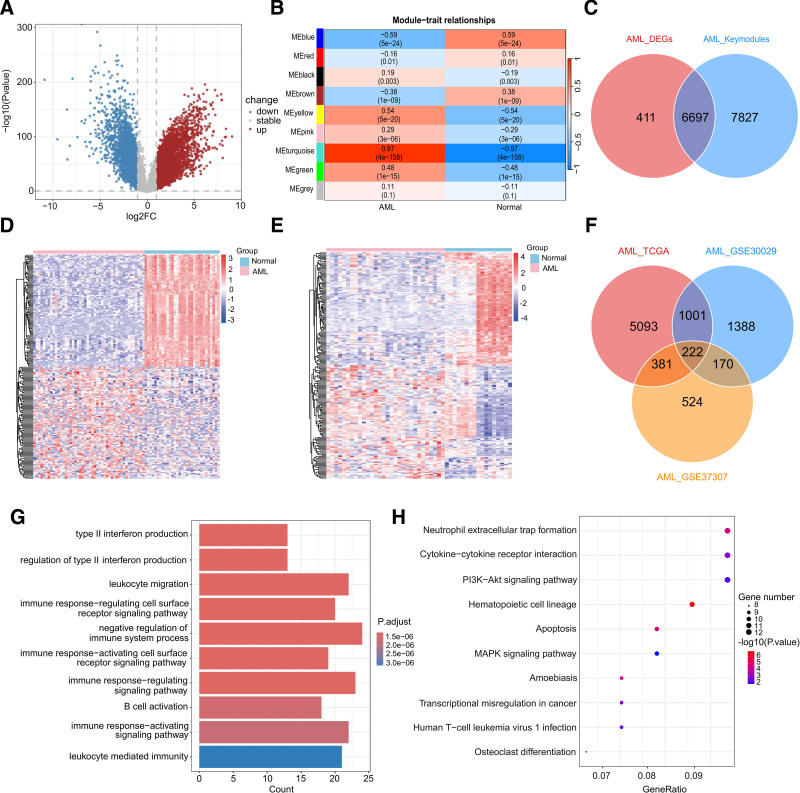
Integration of AML datasets and differential expression analysis. (A) Volcano plot presenting the DEGs between AML patients and normal samples. Upregulated genes are indicated by red dots, while downregulated genes are indicated by blue dots. (B) Heatmap illustrating the relationship between co-expression modules and disease status. The correlation between model Eigen genes and AML traits (top) is depicted, along with corresponding *P* values (bottom). (C) Venn diagram depicting the intersection of key modules and DEGs. A total of 6697 key genes in TCGA-AML were identified. (D, E) The heatmap displaying the top 100 most significantly upregulated or downregulated DEGs in GSE30029 dataset (D) and GSE37307 dataset (E) after difference analysis. (F) Venn diagram depicting the intersection of GSE30029 and GSE37307 datasets and TCGA-AML cohort. (G, H) The GO (G) and KEGG (H) results for key genes of AML. AML = acute myeloid leukemia, DEG = differentially expressed gene, GO = gene ontology, KEGG = Kyoto Encyclopedia of Genes and Genomes, TCGA = the cancer genome atlas.

Similarly, we identified 7108 DEGs in the TCGA-AML cohort (**Fig. 2A**). The WGCNA algorithm was applied, resulting in 9 co-expression modules through average hierarchical linkage. The turquoise module was identified as a key co-expression cluster related to AML (**Fig. 2B**). The overlap between the 2 methods was 6697 genes (**Fig. 2C**). To further narrow down the candidates, 2 GEO datasets (GSE30029 and GSE37307) were used to identify DEGs in AML. Heatmaps display the top 100 dysregulated DEGs in each dataset (**Fig. 2D and E**). Finally, 222 key genes were identified between the 2 GEO datasets and the TCGA-AML dataset (**Fig. 2F**; Table S2, http://links.lww.com/BS/A111). We also performed enrichment analysis to investigate the biological behavior of these key genes. The top 3 GO terms were type II interferon production, regulation of type II interferon production, and leukocyte migration (**Fig. 2G**; Table S5, http://links.lww.com/BS/A114). KEGG analysis of key AML genes revealed their involvement in neutrophil extracellular trap formation, cytokine–cytokine receptor interactions, and the phosphatidylinositol 3-kinase (PI3K)-protein kinase B (Akt) signaling pathway (**Fig. 2H**, Table S6, http://links.lww.com/BS/A115). The key genes in both diseases were involved in common pathways like immune-related and MAPK pathways. Our findings highlight the specific gene functions that may integrate these 2 diseases at the functional level.

### 3.3. The shared genes in PV and AML

**Figure 3.**
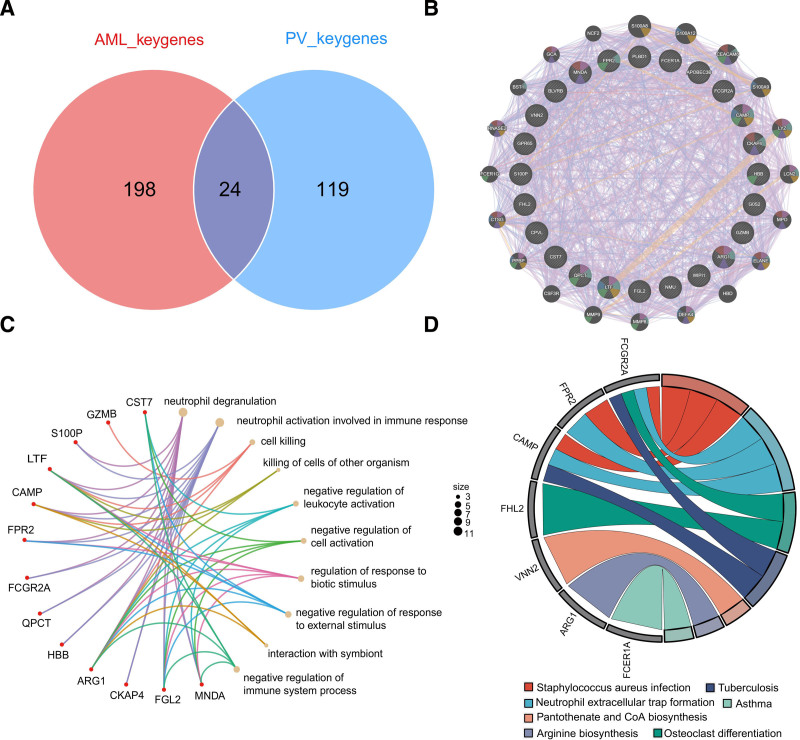
Comprehensive analysis of genes shared by PV and AML. (A) The intersection of key genes in PV and AML resulted in 24 shared genes. (B) PPI network for the obtained 24 shared genes constructed using GeneMANIA. (C) The circular network diagrams depicting the GO-BP enrichment results for the shared genes. (D) KEGG enrichment results and correlations of representative genes and pathways. AML = acute myeloid leukemia, GO-BP = gene ontology-biological process, KEGG = Kyoto Encyclopedia of Genes and Genomes, PV = polycythemia vera.

After intersecting the key genes of PV and AML, 24 shared genes were identified (**Fig. 3A**). The PPI network illustrated interactions between these 24 genes (**Fig. 3B**). Further investigation of the roles of these genes was conducted using GO and KEGG enrichment analyses. In the BP terms of the GO analysis, the top 3 terms were neutrophil degranulation, neutrophil activation involved in the immune response, and negative regulation of the immune system (**Fig. 3C**). KEGG analysis indicated the involvement of these genes in neutrophil extracellular trap formation (**Fig. 3D**).

### 3.4. Identification of hub genes through machine learning

**Figure 4.**
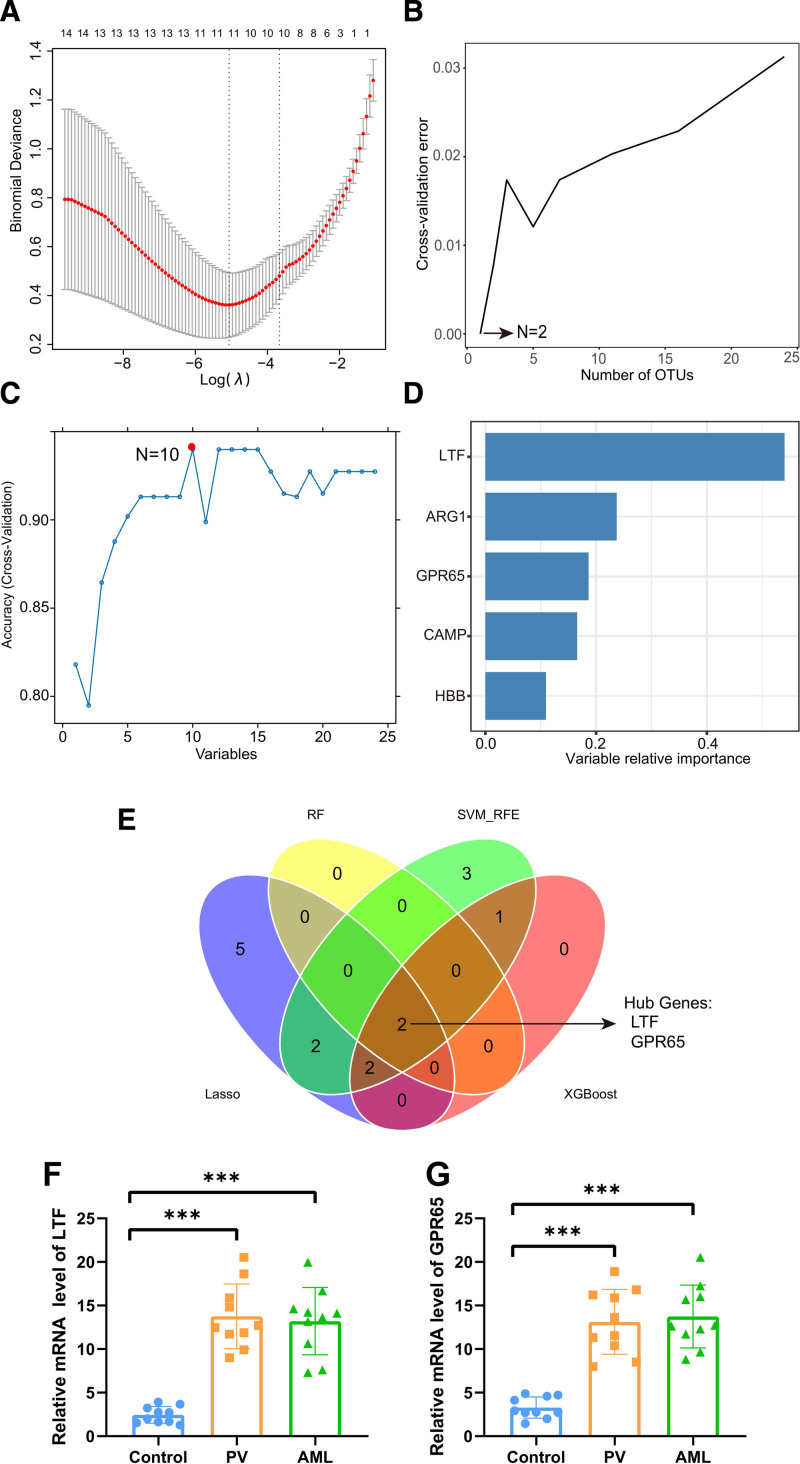
Screening and validation of potential diagnostic markers for AML-related PV using machine-learning approach. (A) Relative change of binomial deviance versus log(λ) plot. The plot demonstrates the variation in binomial deviance as the number of genes included in the model increases. It shows that when the number of genes is 11, the binomial deviance is at its lowest, indicating optimal model performance. (B) Cross-validation curve of the RF algorithm. The curve illustrates the relationship between the number of features retained and the error rate. The plot suggests that the RF algorithm achieves the smallest error when retaining 2 features. (C) SVM-RFE algorithm selected 10 diagnostic biomarkers with the highest accuracy. (D) The XGBoost algorithm is utilized to screen biomarkers and illustrate the relative importance of the top 5 genes. (E) The intersection of genes identified by 4 machine-learning methods yields the most significant 2 potential diagnostic biomarkers (LTF and GPR65) in AML-related PV. (F) RT-qPCR results demonstrating elevated mRNA levels of LTF in clinical samples from both PV and AML patients. (G) RT-qPCR results indicating elevated mRNA levels of GPR65 in clinical samples from both PV and AML patients. ***P < .001. AML = acute myeloid leukemia, GPR65 = G protein-coupled receptor 65, LTF = lactoferrin, OTU = operational taxonomic unit, PV = polycythemia vera, RF = random forest, RT-qPCR = reverse transcription-quantitative polymerase chain reaction.

Given that leukemic transformation is the primary cause of death in patients with PVs,^22^ we aimed to develop a model to distinguish AML-related PV based on shared genes. Using LASSO regression, we identified 11 candidate genes (**Fig. 4A**). The RF algorithm identified 2 genes with the lowest cross-validation error (**Fig. 4B**), whereas the SVM-RFE method identified 10 genes with the highest cross-validation accuracy (**Fig. 4C**). The XGBoost algorithm identified the top 5 features as LTF, ARG1, GPR65, CAMP, and HBB (**Fig. 4D**). Finally, LTF and GPR65 emerged as robust biomarkers across all 4 machine-learning methods (**Fig. 4E**). To validate the robustness of our comprehensive bioinformatic analysis, we assessed the expression levels of these 2 hub genes in clinical samples. RT-qPCR results confirmed that the 2 hub genes were significantly upregulated in patients with both PV and AML (**Fig. 4F, G**).

### 3.5. Generation of diagnostic model

**Figure 5.**
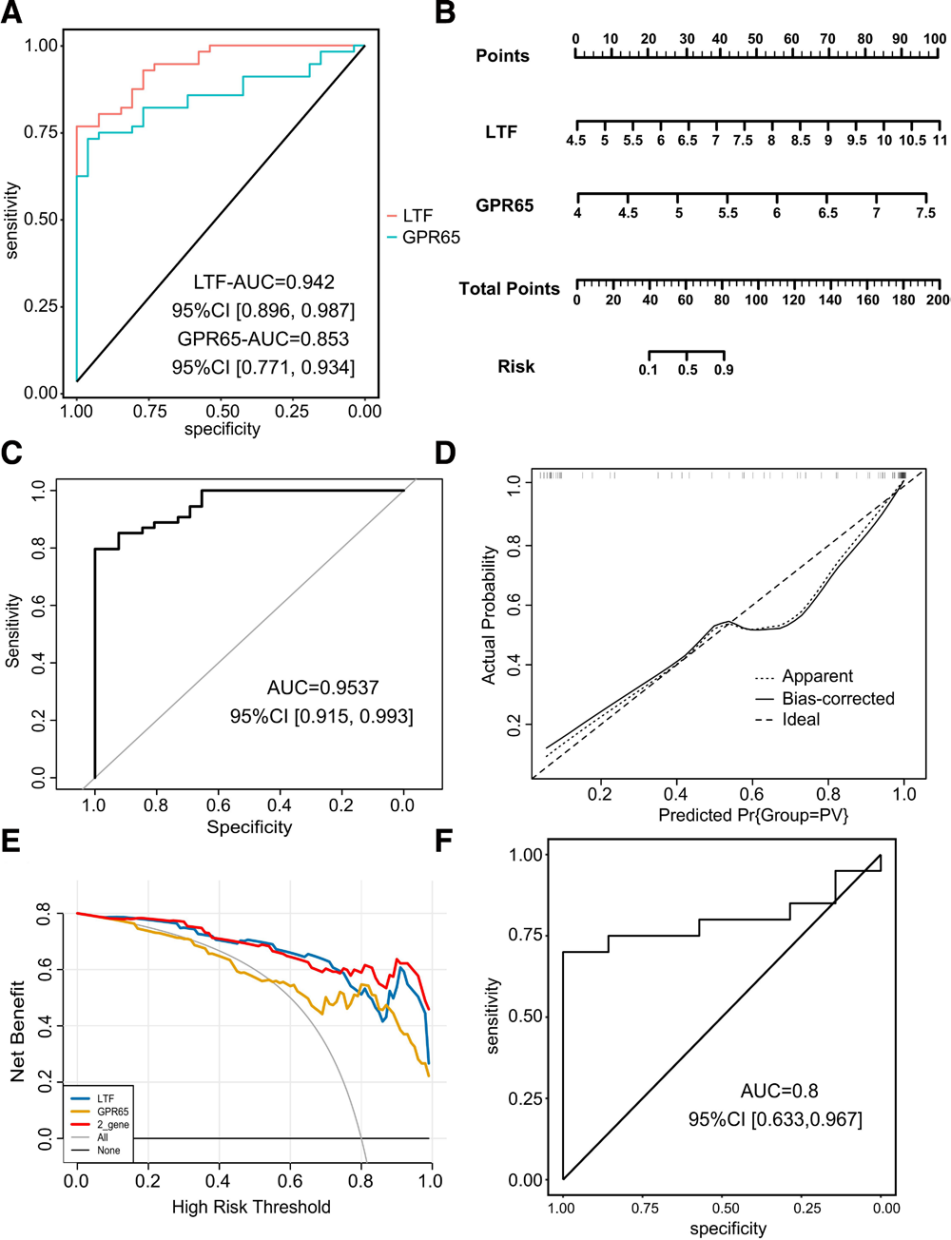
Establishment and evaluation of diagnostic nomogram model. (A) ROC curves illustrating the performance of 2 candidate biomarkers. (B) Nomogram model developed by incorporating LTF and GPR65, providing a visual tool for diagnostic prediction. (C) ROC curves assessing the diagnostic performance of the nomogram model. (D) Calibration curves evaluating the performance of the nomogram model. (E) DCA curves comparing the clinical utility of the nomogram model with single genes. (F) ROC curves demonstrating the performance of the nomogram model in an independent cohort. CI = confidence interval, DCA = decision curve analysis, GPR65 = G protein-coupled receptor 65, LTF = lactoferrin, ROC = receiver operating characteristic.

ROC curves showed that the area under the curve (AUC) for LTF and GPR65 were 0.942 and 0.853, respectively (**Fig. 5A**). Subsequently, we constructed a nomogram model for AML-associated PV based on the expression levels of these 2 genes (**Fig. 5B**). The AUC value of the nomogram model was 0.9537, indicating high accuracy (**Fig. 5C**). Subsequently, calibration curves were constructed to validate the predictive efficiency of the model (**Fig. 5D**), and the DCA curve illustrated the greater benefit of the model compared to that of a single gene (**Fig. 5E**). The ROC curve of the external cohort verified the effectiveness of the nomogram (AUC = 0.8) (**Fig. 5F**).

### 3.6. Identification of candidate small-molecular compounds for AML-associated PV treatment

**Figure 6.**
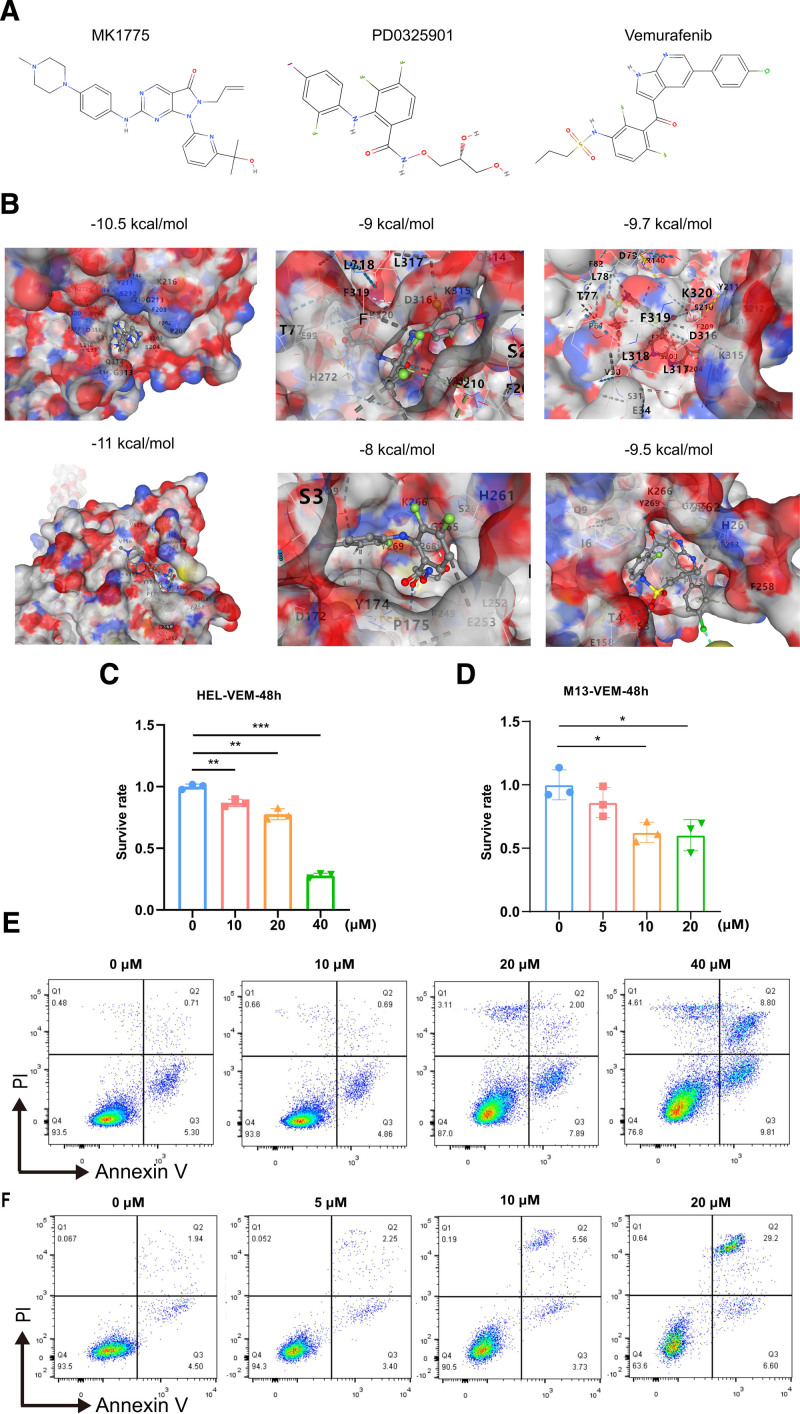
Prediction of potential small-molecule compounds for the treatment of PV using cMAP analysis. (A) Structural formulas of 3 small-molecule drugs have been determined, which can directly display their chemical structures. (B) Schematic diagram of molecular docking structure of Hub genes to 3 small-molecule drugs. (C) Bar plot of small-molecule drug vemurafenib inhibiting HEL cell line proliferation. (D) Bar plot of small-molecule drug vemurafenib inhibiting M13 cell line proliferation. (E, F) Small-molecule drug vemurafenib promotes apoptosis of HEL cell line (E) and M13 cell line (F). *P < .05, **P < .01, ***P < .001. cMAP = connectivity map, PV = polycythemia vera.

To explore potential drugs to treat patients with PV at risk of progression to AML, we performed drug prediction based on hub gene expression. Based on the cMAP database, we identified 3 potential small-molecule drugs (vemurafenib, MK-1775, and PD-0325901) that might modulate the expression of these genes in PV. Furthermore, we performed molecular docking of the 3 drugs using a hub. The results indicated these 3 drugs could significantly bind to the hub genes (**Fig. 6A**). As the effects of MK-1775 and PD-0325901 on PV and AML have been reported,^23–25^ we selected vemurafenib for further studies. CCK8 assays showed that vemurafenib significantly inhibited the proliferation of PV and AML cells (**Fig. 6C and D**). Flow cytometry results showed that vemurafenib promoted apoptosis in both cell lines (**Fig. 6E and F**).

### 3.7. Microenvironment analysis in PV

**Figure 7.**
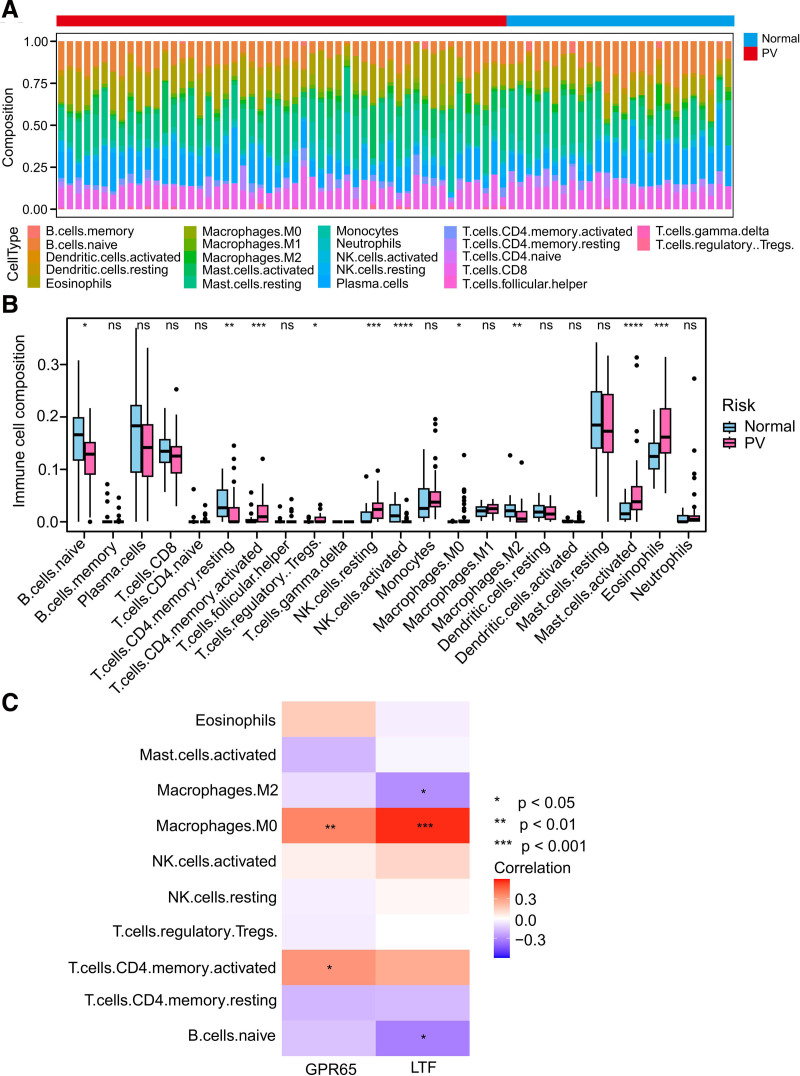
Analysis of immune cells infiltration in PV. (A) The proportion of various immune cells between the PV and control groups. (B) Comparison of 22 immune cell types between the PV and control normal groups. (C) Spearman correlation depicting the association between 2 genes and differential immune cells observed between PV and normal samples. *P < .05; **P < .01; ***P < .001; ****P < .0001. NK = natural killer, PV = polycythemia vera.

Emerging evidence suggests that immune dysregulation plays a pivotal role in the onset and progression of PV.^26,27^ Furthermore, enrichment analysis of the 24 shared genes indicated their involvement in immune-related functions. Consequently, we used the CIBERSORT algorithm to investigate the characteristics of the immune cells in patients with PV. The bar plot illustrates the composition of the immune microenvironment in each patient with PV (**Fig. 7A**). Through differential analysis, we observed alterations in the immune patterns of patients with PV compared with normal samples (**Fig. 7B**). For example, patients with PV exhibit a lower infiltration of activated natural killer (NK) cells and a higher infiltration of M0 macrophages. In addition, we explored the correlation between hub genes and immune cells and found that both LTF and GPR65 were positively correlated with M0 macrophages (**Fig. 7C**).

### 3.8. Single-cell RNA-sequencing (scRNA-seq) data analysis of AML

**Figure 8.**
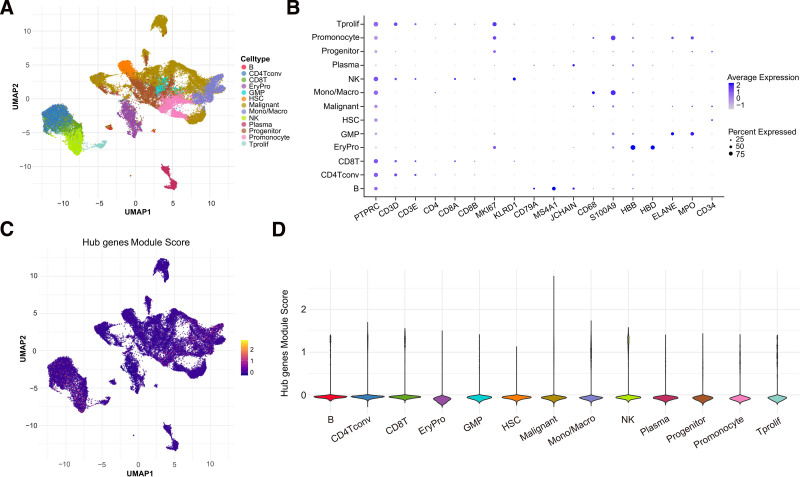
Single-cell data analysis for acute myeloid leukemia. (A) UMAP plot of sc-RNAseq data showed 13 distinct clusters. (B) Dot plot of marker gene expression in different key cell types. (C) UMAP plot of hub gene expression in different cell clusters. (D) Violin diagram shows the expression of hub gene (LTF, GPR65) in different cell clusters. AML = acute myeloid leukemia, GMP = granulocyte-monocyte progenitors, GPR65 = G protein-coupled receptor 65, HSC = hematopoietic stem cells, LTF = lactoferrin, NK = natural killer.

To investigate the association between hub genes and the tumor microenvironment (TME) in AML, we comprehensively analyzed the AML scRNA-seq dataset, GSE116256. Using canonical markers, we identified 13 cell types: B cells, conventional CD4+ T cells, CD8+ T cells, erythroid progenitor cells, granulocyte-monocyte progenitors (GMP), hematopoietic stem cells (HSC), malignant cells, monocytes/macrophages, NK cells, plasma cells, progenitor cells, promonocytes, and proliferative T cells (**Fig. 8A and B**). Using the AddModuleScore algorithm, hub gene scores across diverse cell populations were calculated. Our findings revealed that proliferative T cells, monocytes/macrophages, and NK cells exhibited relatively high scores (**Fig. 8C and D**), suggesting the potential influence of hub genes on the microenvironment.

## 4. DISCUSSION

PV and AML are common hematological malignancies that seriously affect the quality of life of patients. Despite the reported associations between these diseases,^7^ their underlying mechanisms remain elusive. The transformation of PV into AML poses a severe threat to survival, necessitating effective drug interventions.^28^ Therefore, extensive research into shared internal workings is vital. The identification of new molecular markers for the diagnosis of AML-related PV has the potential to enhance its clinical management.

In this study, we successfully identified 24 key genes shared between PV cells and AML cells. Enrichment analysis revealed their involvement in immune-related processes, particularly neutrophils. Recent research has highlighted the abnormal activity of key cells in the immune system as a characteristic of MPN.^29,30^ Over 90% of patients exhibit JAK2 activating mutations. The JAK/STAT pathway, which is responsible for cytokine signal transduction, plays a crucial role in key immune system pathways and cytokine production in mature immune cells.^31^ Studies have identified increased monocyte levels as an independent poor prognostic factor for overall survival in patients with PV.^27^ In addition, considering the role of the microenvironment in AML progression, angiogenesis, metastasis, and drug resistance,^32,33^ exploration of neutrophils and their associated immune signaling has emerged as a promising avenue for therapeutic strategies in patients with both PV and AML.

We used 4 common machine-learning methods to identify LTF and GPR65 as potential diagnostic markers of PV and AML. The upregulation of these genes was validated using an external dataset and clinical samples, reinforcing their promising application prospects. LTF, a member of the transferrin gene family, has been identified in secondary granules of neutrophils. Previous studies have highlighted its significance as a primary iron-binding protein in milk and human secretions, contributing to the nonspecific immune system.^34,35^ Two previous bioinformatics studies implicated LTF as a key gene in essential thrombocythemia and primary myelofibrosis.^36,37^ GPR65 is a G-protein-coupled receptor associated with immune regulation in various studies.^38–40^ Cao et al^41^ reported its potential targeting by the oncogenic factor miR-17 in AML, suggesting a plausible involvement of GPR65 in AML pathogenesis. LTF plays a significant role in regulating immune responses by stimulating the activity of NK cells, macrophages, and neutrophils while balancing cytokine production to reduce inflammation.^42,43^ Similarly, GPR65 is crucial to the immune system, functioning as a pH sensor and regulating immune cell functions. It influences the behavior of various immune cells, including T cells, macrophages, and dendritic cells.^44,45^ The correlation analysis in Figure 7C revealed that both LTF and GPR65 levels were significantly positively correlated with M0 macrophages. This observation suggests that LTF and GPR65 influence the immune dynamics, particularly through their effects on M0 macrophages. In addition, it has been found that GPR65 is upregulated in tumor-associated macrophages (TAMs) under obesity conditions, promoting tumor growth and inhibiting inflammatory responses, which is consistent with our findings.^46^ However, the precise roles of LTF and GPR65 in the transformation of PV cells into AML require further investigation.

Microenvironmental analysis revealed notable differences in the immune composition between patients with PV and normal samples. Lower levels of activated NK cells have been observed in patients with PV. A relevant study indicated that ruxolitinib, an approved treatment for PV, adversely affects NK cell function in patients with MPN.^47^ As the landscape of immunotherapy continues to evolve, there is a need to develop drugs that modulate the PV immune system and potentially mitigate the side effects associated with first-line treatment.^48^ This insight underscores the importance of considering immune dynamics in treatment strategies for PV, prompting the exploration of novel therapeutic avenues that balance efficacy and immune system integrity. Understanding the relationship between AML and the TME is crucial for understanding tumor progression and treatment resistance.^49,50^ Interactions within the TME provide critical survival signals for AML cells, aiding their proliferation, and protecting them from apoptosis. This supportive environment fosters clonal evolution of AML, favoring malignant cell survival.^51,52^ TME facilitates immune evasion by AML cells, creating an immunosuppressive milieu between regulatory T cells (Tregs) and myeloid-derived suppressor cells (MDSCs). These dynamics underscore the importance of targeting the TME interactions in therapeutic strategies. Potential interventions, including signaling pathway inhibitors and immune checkpoint blockers, show promise in enhancing treatment efficacy.^53–55^

Our study identified 10 potential small-molecule drugs for AML-related PV. Raltegravir, an HIV-1 integrase strand transfer inhibitor, is well tolerated and convenient.^56^ A patient diagnosed with acute promyelocytic leukemia (APL) and HIV achieved complete cytogenetic and molecular remission after treatment with all-trans retinoic acid (ATRA), idarubicin, and raltegravir.^49^ This suggests that the drugs used for APL and antiretroviral treatments may have overlapping effects and could target both the diseases. OM-137 is an Aurora kinase inhibitor known for its ability to inhibit mitosis.^50^ Similarly, Alisertib, another Aurora A inhibitor, demonstrated good tolerability and efficacy combined with induction chemotherapy in patients with AML in a phase I study.^51^ Furthermore, CG-806, a multi-kinase inhibitor, exhibits anticancer activity against AML by simultaneously targeting FLT3, BTK, and Aurora kinases.^52^ Flecainide, a class Ic antiarrhythmic drug, non-selectively blocks the sarcoplasmic reticulum (SR) lumen for cytoplasmic Ca²^+^ release via intracellular calcium release channels, RyR2.^53^ Sheth et al^54^ discovered that AML leukemic stem cells (LSCs) resistant to venetoclax exhibited a more active metabolic state with relatively high calcium levels. Consequently, the combination of venetoclax and flecainide may be beneficial in treating patients with AML. T-0070907, a PPAR receptor antagonist. Several studies have demonstrated the therapeutic potential of PPARα targeting in AML.^49,56^ These drugs deserve further investigation to pave the way for new drug targets for AML treatment.

It is important to acknowledge the limitations of this study. First, it was constrained by the limited volume of data, necessitating validation in a larger cohort for robustness. In addition, exploration of important genes in PV and AML would benefit from further biological experiments to elucidate their potential roles. Finally, the application of LTF and GPR65 warrants direct testing in patients who transition from PV to AML. Furthermore, the potential applications of LTF and GPR65 as biomarkers or therapeutic targets for the conversion of PV to AML require direct clinical testing. This will provide valuable insights into their predictive value and efficacy, ultimately helping provide patients with more nuanced treatments that can be better applied to clinical treatments.

Despite these limitations, our integrative analyses identified molecular connections between PV and AML that have not been previously reported. The nomogram model demonstrated its ability to stratify patients with PVs. LTF and GPR65 have been identified as potential candidates for biomarker monitoring. Our findings lay the foundation for further experimental validation. This study provides novel insights into significantly affecting clinical management and treatment selection, particularly with immunotherapeutic approaches for patients with AML-related PV.

## ACKNOWLEDGMENTS

This study was supported by grants from the National Natural Science Foundation of China (Grant numbers 81920108004, 82270127, and 81800125), the grants from Shenzhen Health Development Research and Data Management Center (Grant numbers sz20230199 and 0868-2344ZD1274F), the Hunan Provincial Natural Science Foundation (Grant numbers 2024JJ3037, 2023JJ30928, and 2025JJ60692), the Changsha Science and Technology Bureau (Grant number kq2208382).

## ETHICAL APPROVAL

Ethical permissions were granted by the institutional review board of The Xiangya Hospital of Central South University. All patients provided their informed written consent to participate in this study.

## AUTHOR CONTRIBUTIONS

J.L., X.-F.H., X.-S.W., and H.G. contributed to the design of the article. H.G., H.L., P.-X.C., S.L., J.M., X.-Y.L., T.G., L.N., S.-J.S., X.-J.X., and X.-F.H. collected and analyzed the data. H.L., C.-Y.Y., and H.G. performed the experiments. J.L., H.G., X.-S.W., and H.L. wrote and revised the manuscript. All authors read and approved the final manuscript.

## Supplementary Material


